# Comparison of two high dose rate intracavitary brachytherapy regimens in treatment of cervical cancer: a preliminary report

**DOI:** 10.1007/s12672-023-00646-x

**Published:** 2023-03-29

**Authors:** Abhishek Krishna, AG Hasib, Donald Fernandes, M. S. Athiyamaan, Sandesh Rao, Sharaschandra Shankar, Mohsina Ali, Hepsiba Priyadarshini, Maria Sophia, CH Shridhar, Seby George, Amrutha Babu, Sourjya Banerjee, Johan Sunny, Challapalli Srinivas, Dilson Lobo

**Affiliations:** 1 Department of Radiation Oncology, Kasturba Medical College, Mangalore, Manipal Academy of Higher Education, Manipal, Mangalore, India; 2 Department of Radiation Oncology, Father Muller Medical College, Mangalore, India; 3 Sultan Qaboos University, Muscat, Oman

**Keywords:** Carcinoma cervix, Brachytherapy, Intracavitary radiotherapy

## Abstract

**Background:**

To assess and compare the local control and toxicities between HDR Intracavitary Brachytherapy with 7.5 Gy and 9 Gy per fraction after EBRT in treatment of carcinoma cervix.

**Methodology:**

A total of 180 patients were randomly assigned to 2 arms. Arm A received HDR intracavitary brachytherapy with a dose of 7.5 Gy per fraction, 1 fraction per week for 3 fractions and Arm B received 9 Gy per fraction, 1 fraction per week for 2 fractions. Patients were evaluated on follow up for assessment of local control and toxicities.

**Results:**

The median follow up was 12 months (6–18 months). In arm A 89% of the patient had complete response and 11% had recurrence or metastasis. In arm B 93% of the patient had complete response and 7% had recurrence or metastasis. Grade 2/3 diarrhoea was seen in 4.4% of patients in Arm A and in 7.7% in Arm B. Grade 2/3 proctitis was seen in 3.3% of patients in 7.5 Gy arm and in 6.6% in 9 Gy arm. One patient in each arm had grade 1 haematuria. The overall duration of treatment was significant lower in Arm B compared to Arm A (59 days vs 68 days, p = 0.01).

**Conclusion:**

The result of this clinical study shows that Intracavitary brachytherapy with a dose of 9 Gy per fraction is non inferior to other schedules in term of local control and does not result in increased toxicity.

## Introduction

The number of new cancer cases worldwide has grown to 18.1 million [1]. Cervical cancer is the fourth most prevalent cancer in females across the globe, and the ninth most prevalent cancer overall [1]. Cervical carcinoma is the second most frequent cancer among Indian women [2, 3]. Majority of the cases of carcinoma cervix present as locally advanced disease. The mainstay of treatment for advanced cancers of the cervix is external beam radiation (EBRT) with concomitant cisplatin injection followed by brachytherapy [4–7]. High dose-rate (HDR) Intracavitary Brachytherapy (ICBT) for malignancy of the cervix is now well established because of the numerous advantages it provides. Significantly Short treatment duration leads to fewer hospital stays and increased patient comfort. HDR Brachytherapy has enabled the combination of EBRT with brachytherapy, resulting in a much-reduced total treatment time and improved tumour control [8, 9].

Despite a good amount of literature and studies on the efficiency of HDR brachytherapy, the optimal treatment time, radiation dosage, and fractionation schedule are still unknown. There are only very few studies based on the optimal fractionation and dosage in intracavitary brachytherapy in carcinoma cervix. Individual fraction sizes of less than or equal to 7.5 Gy in 4 to 8 fractions, depending on the dose per fraction, have been suggested by the American Brachytherapy Society (ABS). However, these suggestions come with a warning that the suggestion has not been extensively evaluated [9].

In comparison to ABS, studies have shown that HDR Intracavitary Brachytherapy is safe and effective when the dosage per fraction is greater than 7.5 Gy [8–11]. As External Beam Radiotherapy for Carcinoma cervix spans over 5–6 weeks, lowering the total number of fractions of Brachytherapy leads to a significant reduction in the overall treatment time. The aim of this study was to assess HDR Intracavitary Brachytherapy in cervical cancers (9 Gray in each fraction for two fractions vs. 7.5 Gray in each fraction, for three fractions) in terms of disease response and toxicities.

## Methods

This was a single institution randomised study comparing two different dose schedules of brachytherapy in the treatment of cervical cancer. Eligibility criteria included patients with histologically confirmed cervical cancer, completed Pelvic EBRT 46–50 Gy in 23–25 fractions, deemed suitable for intracavitary application, Karnofsky performance status of more than or equal to 70 and adequate hematological and renal parameters. Exclusion criteria were patients with metastasis, unsuitable for Intracavitary application, other malignancy and with previous radiotherapy to pelvis.

**Fig. 1:**
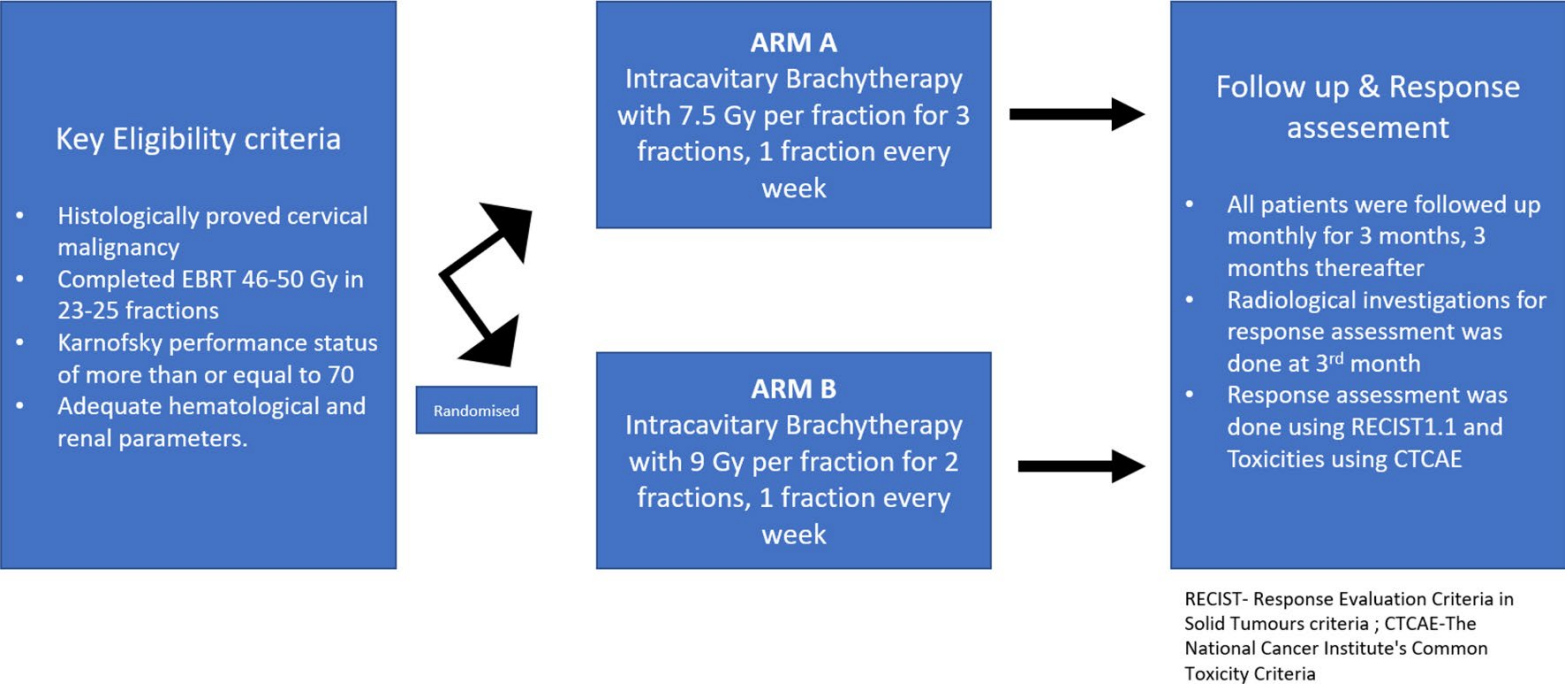
Patient and Study Schema

All patients underwent clinical examination to ascertain the suitability of Intracavitary application after the completion of EBRT. All eligible participants were randomly assigned to one of two arms at random: Arm A- HDR Intracavitary brachytherapy 7.5 Gy in 3 fractions, 1 fraction a week which was the institution standard and to ARM B—HDR Intracavitary brachytherapy 9 Gy in 2 fractions, 1 fraction a week (Fig. 1).

The institutional protocol utilized a dose fraction of 7.5 Gy, while a dose fraction of 9 Gy was selected based on the findings of multiple studies available in the literature which a demonstrated comparable to superior local control with higher doses per fractionation, despite a reduction in the overall dose of brachytherapy administered [8, 10–16].

**Fig. 2:**
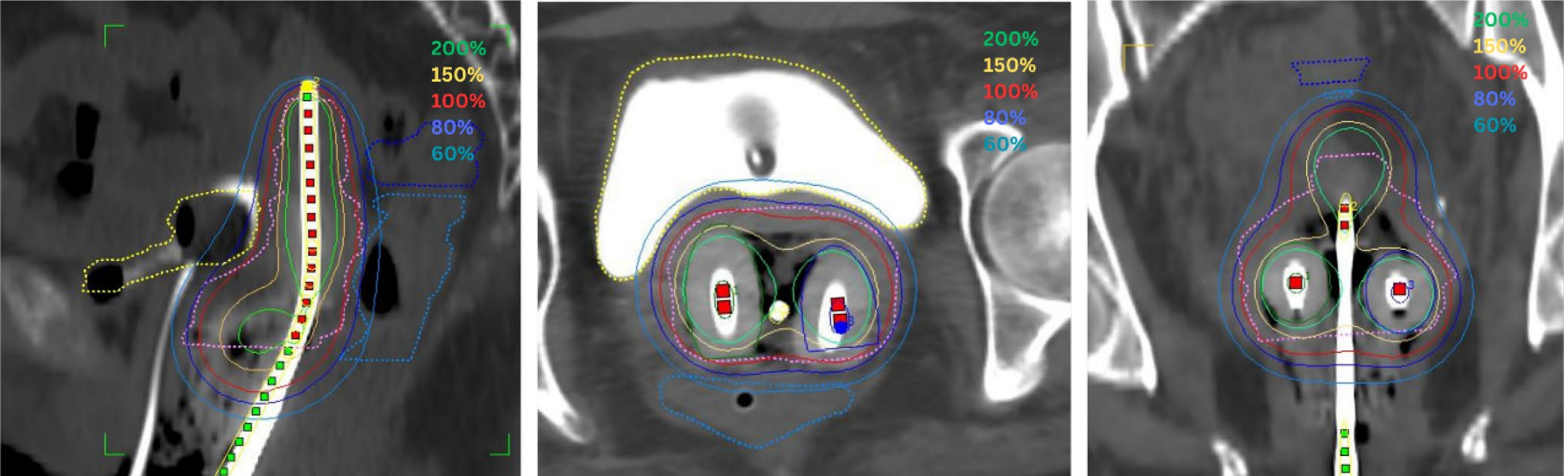
CT scan with Brachytherapy applicator in situ

The Intracavitary applicator insertion was done in the Operation Theatre under spinal anaesthesia. Optimal vaginal packing was done. Modified Fletcher Suite Tandem and Ovoid applicator were used for brachytherapy. Brachytherapy treatment planning was done on a CT scan. Image scanning with 3.0 mm slice thickness extending from the iliac crest was performed during each fraction with the applicator in place (Fig. 2).

High-risk clinical target volume (HR-CTV) was defined as the volume bearing the highest risk for recurrence. Contouring of HR-CTV, IR-CTV and at-risk organs were done as per GEC ESTRO guidelines [17]. HR-CTV D90 was defined as the minimum dose received by 90% of the volume of the HR-CTV; IR-CTV D90 was defined as the minimum dose received by 90% of the volume of the IR-CTV. D2cc of the organ was the minimum dose received by maximally irradiated 2 cc volume.

Optimization was performed, by accounting the EBRT dose to result in a total of equivalent dose in 2 Gy fractions (EQD2) dose volume histogram (DVH) constraints of 80- 90 Gy (α/β = 10) for HR-CTV while keeping minimum dose to the most exposed 2cm3 volume (D2cc) of bladder and rectum/sigmoid to total EQD2 90 Gy (α/β = 3) and 75 Gy (α/β = 3) respectively.

For dosimetry evaluation, the following dose parameters were recorded: The minimum dose covering 90% of HR-CTV volume—D90 HR-CTV; minimum dose covering 90% of IR-CTV volume—D90 IR-CTV, D2cc for rectum and D2cc for bladder.

Evaluation of local control was done on a clinical basis before each session of ICBT and monthly after the completion of treatment. Radiological investigations were done at 3^rd^ month for response evaluation and thereafter as and when deemed necessary. The Response Evaluation Criteria in Solid Tumours criteria were used to assess the patient's response [18]. The National Cancer Institute's Common Toxicity Criteria version 5 was used to assess and grade toxicity [19]. Chi-square test and Student’s *t*-test were used for data analysis. A p-value of less than 0.05 was considered to indicate statistical significance. Statistical analysis was done using the Statistical Package for Social Sciences, version 22.

## Results

### Patient characteristics

One hundred and ninety individuals with histopathological confirmed cervical cancer were included in the study over the study period. Patients were assigned to one of two arms using a computer-generated random number table. Figure 1 depicts the randomization and allocation of participants into two arms. Schema of randomization and allocation of patients into two arms were shown in Fig. 1. Five patients in each arm were excluded from the final analysis due to incomplete treatment and lost to follow up. The remaining total number of 180 patients of 90 in each arm was analysed in this study.

The median age of patients was 56 years in Arm A and Baseline patient characteristics were balanced in both the arms. The most common stage at diagnosis was IIB in both the arms. Most common histology was squamous cell carcinoma in both arms. The demographic and tumour characteristics are outlined in Table 1.

**Table 1:** Patient and baseline characteristics

Patient characteristics	Arm A (7.5 Gy) n = 90 (%)	Arm B (9 Gy) n = 90 (%)
Age in years (median)	56	55
Stage at diagnosis		
IB1	2 (2.2%)	1 (1.1%)
IB2	1 (1.1%)	2 (2.2%)
IIA	7 (7.7%)	8 (8.9%)
IIB	43 (47.6%)	41 (45.5%)
IIIA	6 (6.7%)	5 (5.6%)
IIIB	19 (21%)	20 (22.2%)
IIIC1	9 (10%)	7 (7.7%)
IIIC2	2 (2.2%)	2 (2.2%)
IVA	4 (4.4%)	4 (4.4%)
Histology		
Squamous cell carcinoma	81 (90%)	81 (90%)
Adenocarcinoma	8 (8.9%)	6 (6.7%)
Adenosquamous carcinoma	1 (1.1%)	1 (1.1%)
Clear cell carcinoma	0 (0%)	2 (2.2%)
Whole pelvis EBRT dose		
50 Gy in 25 fractions	90 (100%)	90 (100%)
Concurrent chemotherapy agent		
Cisplatin	82 (91.1%)	81 (90%)
Carboplatin	6 (6.7%)	7 (7.8%)
No chemotherapy	2 (2.2%)	2 (2.2%)
Average chemotherapy cycles	4	4
Total duration of treatment- EBRT + brachytherapy (mean days)	68	59

### Treatment characteristics

All patients received a dose of 50 Gy in 25 fractions to pelvis with EBRT. Concurrent chemotherapy with weekly cisplatin were administered to 91% of patients in ARM A and 90% of patients in ARM B. Patients received an average of 4 cycles of chemotherapy. The mean total duration treatment, from the start of EBRT till the day of last brachytherapy was 68 days in ARM A and 59 days in ARM B. The median follow-up of all patients was 12 months (6–18 months).

The results of the dosimetric parameters for both groups are listed in Table 2. The total EQD2 from both EBRT and Brachytherapy for tumours and OAR was ensured to fulfil the DVH constraints objectives set.

**Table 2:** Dosimetric parameters of Tumour Volume and Organs at Risk

Parameters	Brachytherapy		EBRT + Brachytherapy	
	7.5 Gy arm EQD2	9 Gy arm EQD2	7.5 Gy arm EQD2	9 Gy arm EQD2
HR-CTV D90_α/β=10_	39.3 ± 5.3	30 ± 3.9	89.4 ± 5.3 Gy_10_	79 ± 4.1 Gy_10_
IR-CTV D90_α/β=10_	15.29 ± 3.9	10.1 ± 4.2	65.29 ± 3.9 Gy_10_	60 ± 4.1 Gy_10_
Rectum D2cc_α/β=3_	17.4 ± 5.5	11.4 ± 4.2	67.4 ± 5.5 Gy_3_	62.4 ± 4.2 Gy_3_
Bladder D2cc_α/β=3_	23.4 ± 6.5	13.3 ± 4.3	73.4 ± 6.5 Gy_3_	63.3 ± 4.3 Gy_3_

*HR-CTV* high risk clinical target volume, *IR-CTV* intermediate risk clinical target volume, *EQD2* equivalent total doses in 2-Gy fraction

### Local control and treatment failures

**Fig. 3:**
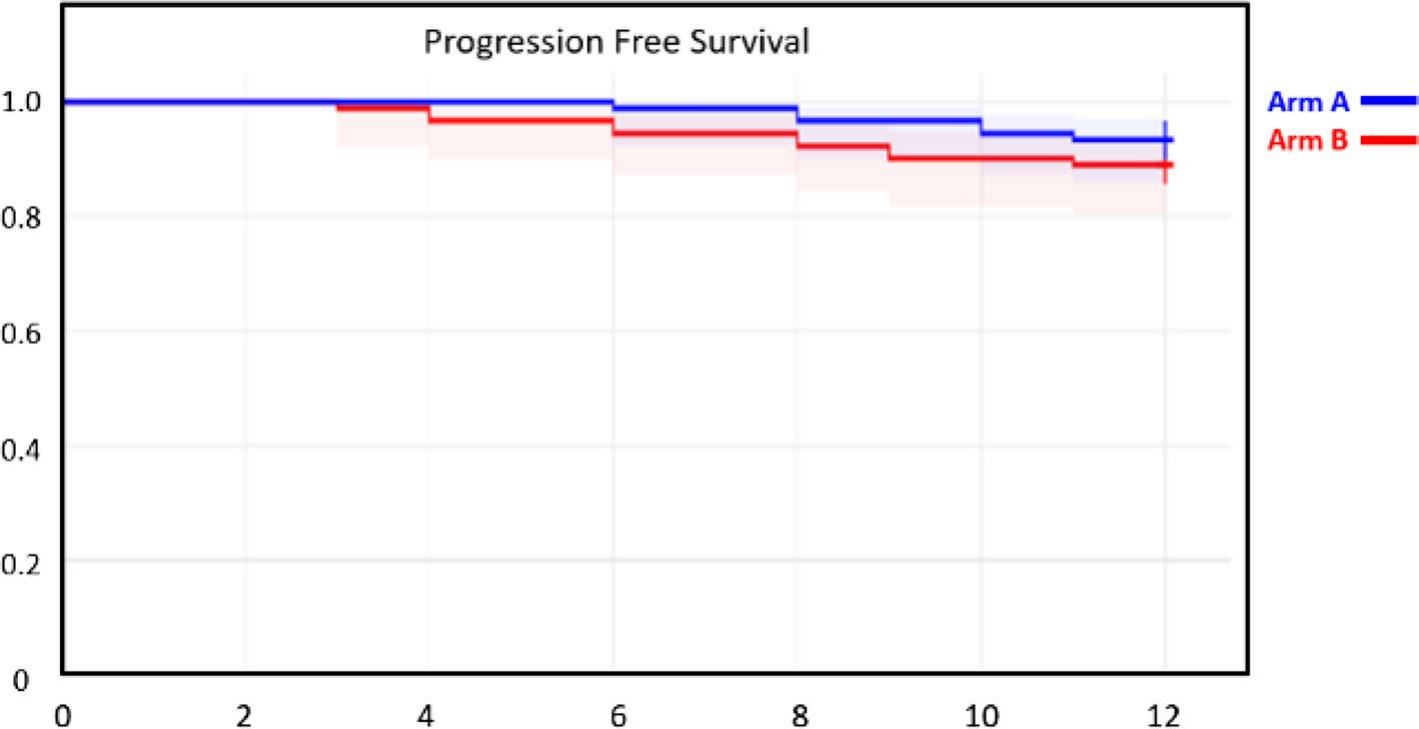
Progression Free Survival in both the arms

In arm A 80 patients had a complete response with 6 patients having locoregional recurrence and 4 patients having distant metastasis. Among the 6 patients with locoregional recurrence, 4 patients had local only and 2 patients has local and nodal recurrence. All patients were deemed inoperable for salvage surgery due to the disease extent and performance status of the patients. Among the 4 patients with distant metastasis, 3 had liver metastasis and 1 had supraclavicular metastasis. In arm B, 84 patients had a complete response with 3 patients having a locoregional recurrence and 3 patients having distant metastasis. Among the patients with locoregional metastasis 1 patient had local and 2 had local and nodal metastasis. Among the patients with distant metastasis 2 patients had liver and 1 patient had supraclavicular metastasis. The PFS at 12 months was 89% in Arm A and 93% in ARM B (p = 0.28) (Fig. 3).

### Toxicities

The D2cc to the bladder per session of brachytherapy was 3.42 ± 1.27 Gy in patients of Arm A and 3.79 Gy ± 1.14 in Arm B. The total EQD2 to the bladder from brachytherapy was 23.4 ± 6.5 Gy in Arm A and 13.3 ± 4.3 Gy in Arm B. The D2cc to the rectum per session of brachytherapy was 3.72 ± 1.08 Gy in Arm A and 4.38 ± 1.14 Gy in Arm B. The total EQD2 to the rectum from brachytherapy was 17.4 ± 5.5 Gy in Arm A and 11.4 ± 4.2 Gy in Arm B as shown in Table 2. In arm A, 7 (7.8%) patients had vomiting which was of Grade 1. One patient each had grade 2 and grade 3 vomiting. None had grade 4 or above vomiting. In Arm B, 5 patients (5.6%) suffered grade 1 vomiting. No one had vomited in a grade 2 or above. There was no statistical difference in both the arms in terms of vomiting.

**Fig. 4:**
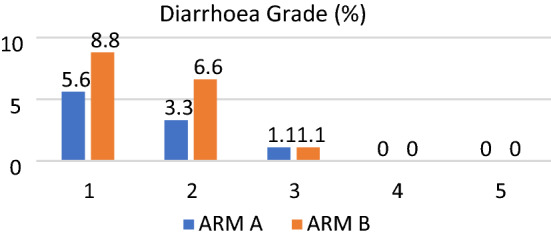
Grade of Diarrhoea

In Arm A 9 patients experienced diarrhoea. Five (5.6%) patients had grade 1 diarrhoea, 3 (3.3%) patients experienced diarrhoea of Grade 2 and 1 (1.1%) patient had grade 3 diarrhoea. In arm B, the incidence of diarrhoea was slightly higher but had no statistical significance. Eight (8.8%) patients experienced grade 1 diarrhoea, 6 (6.6%) patients had diarrhoea of grade 2 and 1 patient experienced grade 3 diarrhoea (Fig. 4). All patients having diarrhoea were treated conservatively with fluids, antidiarrheals and antibiotics whenever required. Two patients with grade 3 diarrhoea were managed on an in-patient basis, and both improved symptomatically over the course of treatment. When the D2cc dose received by the rectum in patients who did not have diarrhoea was compared to the patients who did, it was observed that the patients with diarrhoea received slightly higher dose, particularly in Arm B (4.3 Gy vs 4.9 Gy), although this difference was not statistically significant (Fig. 4).

**Fig. 5:**
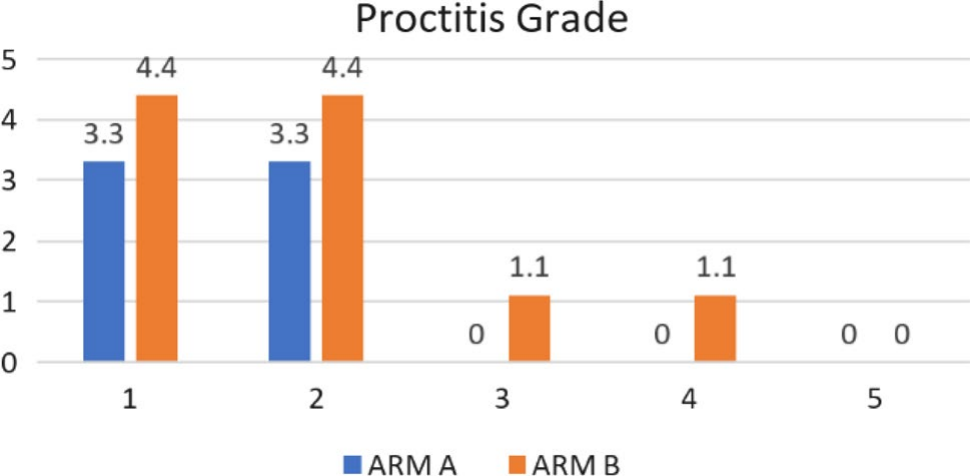
Grade of Proctitis

Grade 1 proctitis was seen in three (3.3%) of patients in Arm A and four (4.4%) of patients in Arm B. Grade 2 proctitis was seen in 3 (3.3%) of patients in Arm A and 4 (4.4%) of patients in Arm B (Fig. 5). None of the patients in Arm A experienced proctitis of grade 3 or 4. Although there was no statistical significance, one patient experienced grade 3 proctitis and one patient experienced grade 4 proctitis. All patients were managed conservatively except one patient with Grade 4 proctitis requiring surgical intervention.

**Fig. 6:**
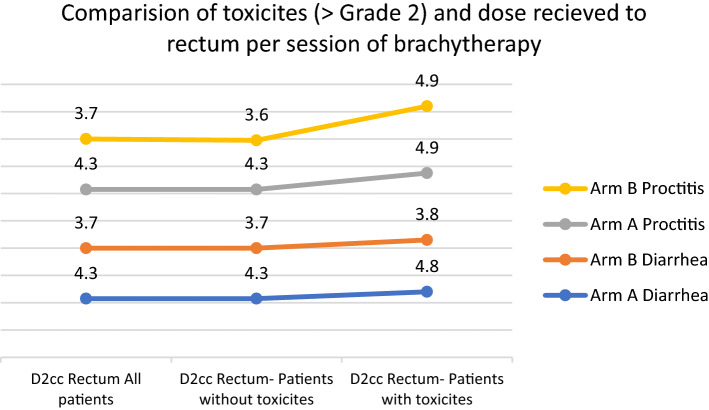
Comparison of toxicities and dose received to rectum per session of brachytherapyA

Patients who had grade > 2 proctitis had received a D2cc rectal dose of 4.9 Gy per session which was higher than the D2cc dose received by patients who did not have proctitis (3.6 Gy). On further analysis, in Arm B, patients who had grade > 2 proctitis had received a D2cc rectal dose of 4.9 Gy per session which was higher than the D2cc dose received by patients who did not have proctitis (4.3 Gy) (Fig. 6).

In arm A, Grade 1 increased urinary frequency was noted in 4 (4.4 percent) patients and Grade 2 in one patient. In arm B, 4 (4.4 percent) individuals had Grade 1 urinary frequency and Grade 2 in one patient reported. Grade 1 haematuria was seen in 1 patient in Arm A and 1 patient in Arm B. None of them had haematuria of grade 2 or higher. There was no statistical difference in terms of genitourinary toxicities between the two arms.

## Discussion

Concurrent chemoradiation with EBRT and brachytherapy is the standard approach in locally advanced cervical cancers. By escalating the dosage following EBRT, BT improves the curative potential and provides a higher dose directly to the tumour while preserving the surrounding critical structures [20]. In the past decade, there has been a shift from LDR intracavitary brachytherapy to HDR Intracavitary brachytherapy due to the various advantages such as significant shorter treatment duration, improved geometric placement, a better patient compliance and reducing patient discomfort and inconvenience [8, 11].

In order to achieve excellent tumour control and reduce the frequency of problems, fractionation and dosage schedules of brachytherapy are critical [8, 12]. If there exists a way to reduce the total number of fractions of radiotherapy, mainly brachytherapy, without compromising the tumour control and toxicities it would, not only reduce the overall treatment time, but also be, more patient compliant. It would also reduce the number of hospital admissions and multiple exposure to anaesthetic agents.

Various studies have evaluated the disease control with utilising higher dose per fraction of brachytherapy as shown in Table 3. In 49 patients treated with ICR of 9–9.4 Gy × 2 fractions, Sood et al. found a 16.3 percent local failure after two years [10]. Ghosh et al. noted patients treated with 9 Gy × 2 fractions had 91.5 percent local control after two years, compared to 88.5 percent in patients treated with 7 Gy × 3 fractions [11]. According to Patel et al., patients who received ICR with 9 Gy × 2 fractions had 81.35 percent local control after 3 years, compared to 65.18 percent in patients who received 6.8 Gy × 3 fractions (p = 0.05) [8]. Novetsky et al. reported a 5-year PFS of 75% in a trial of 77 patients treated with EBRT and ICR with 9 Gy per fraction for 2 fractions [13]. Passi et al., reported a 6-month local recurrence of 3.2% in patients who received ICR with 9.5 Gy × 2 fractions compared to 5.4% in patients who received 7.5 Gy per fraction [14]. In a trial of 604 patients, Hendry et al. found that patients who got 9 Gy × 2 fractions had a 5-year local control rate of 82 percent and patients who received 7 Gy × 4 fractions had a 5-year local control rate of 89 percent (p = 0.72) [15]. In the present study we observed a local control of 89% in 7.5 Gy arm and 93% in 9 Gy arm (p = 0.04).

**Table 3:** Comparison of similar studies in literature

Study & year	Patients	Control arm	Study arm	Results
Patel et al., 2011 [8]	104	6.8 Gy x 3^a^	9 Gy x 2^a^	3 year LC − 65.18% vs 81.35% in study arm
Sood et al., 2002 [10]	49		9–9.4 Gy x 2^a^	3 yr LC—77%
Ghosh et al., 2002 [11]	124	7 Gy x 3^a^	9 Gy x 2^a^	2 year LC—88.5% in control vs 91.5% in study arm
Novetsky et al., 2007 [13]	77		9 Gy x 2^a^	5 year PFS—75%
Passi et al., 2010 [14]	90	7.5 Gy x 3^a^	9.5 Gy x 2^a^	6 month local recurrence—5.4%in control vs 3.2% in Study arm
Hendry et al., 2017 [15]	601	7 Gy x 4^a^	9 Gy x 2^a^	5 year tumour control—88% in Control vs 78% in study arm; 5 year OS 62% in control arm vs 68.3% in study arm

*LC* local control, *PFS* progression free survival, *OS* overall survival

^a^Fractions

Longer overall treatment time (> 56–60 days) have been linked to a greater probability of recurrence [16, 21]. The overall treatment duration was 68 days in Arm A and 59 days in Arm B in our trial. Arm A had 10 failures and Arm B had 6. This signifies the need to complete the course of EBRT and Brachytherapy within the desired timeframe. Undue delay in completion of treatment can lead to treatment failures. There could have been numerous factors that would have caused the delay in completion of treatment. Few patients have toxicities such as mucosal reactions, diarrhoea, neutropenia etc. on completion of CT-RT. Usually a gap of 7 to 10 days is given prior to brachytherapy for the reactions to settle in these patients. Few patients encounter vaginal infections, in whom Brachytherapy is performed only after completion of a course of antibiotics. Other factors that could have contributed to treatment delays are increased patient load, availability of OT and anaesthesia facility and non-adherent to the treatment schedule by the patients.

Local recurrences did occur within the median follow up of 12 months. In arm A 11% of patients had recurrence or distal metastasis and in Arm B, 7% of the patients developed recurrence or distal metastasis.

There have been speculations of higher toxicities with HDR brachytherapy regimens. Certain studies have also shown a slight increased rates of late GI and GU toxicities in patients undergoing ICR with HDR brachytherapy. In contrast to this, there are various advantages of HDR brachytherapy in terms of toxicities.

Various Studies have been done comparing toxicities with higher dose per fraction of BT. Patel et al., in their study reported a slightly higher GI and GU toxicities in the9 Gy arm compared to the 6.8 Gy arm (7.47% vs 3.57%) [8]. Novetsky et al., reported acute toxicities of 47% and late side effects of 6% in their study with ICBT of 9 Gy in 2 fractions [13]. Hendry et al. found that patients who got 9 Gy had a slightly greater rate of toxicities than those who received 7 Gy (7.2 percent vs. 5.3 percent, p = 0.06) [15]. Saptarshi et al., in their study reported a late toxicity of 3% in patients treated with ICR with a higher dose per fraction [16]. Majority of the toxicities occurred during the course of radiation therapy. Diarrhoea and proctitis that occurred during follow up were majorly Grade 1 and Grade 2 and most of them were managed medically. In our study, incidence of rectal and GU toxicities was comparable in 7.5 Gy and 9 Gy arm and there was no statistical difference seen. A slightly higher incidence of Grade 2 and more toxicities were noted in Arm B despite the total dose to rectum being lower than Arm A. This reiterates the fact that dose per fraction is of utmost importance in terms of late toxicities. Under general anaesthesia, HDR insertions allow for successful vaginal packing to shift the essential organs as much as feasible. The packing of the rectum and bladder away from the sources is easier to maintain during the brief procedure, and this benefit more than compensates for the radiobiologic loss of the therapeutic ratio when fewer larger fractions are employed [8]. Patients receiving a higher dose to the rectum encountered a higher grade of toxicities. To reduce toxicities, the dose to the rectum should be kept as low as possible when planning the treatment.

The advantage of utilising fewer fractions is greater patient compliance and convenience. Reduced risk of repeated anaesthetic agent exposures and fewer hospital admissions make this schedule economically effective, which is important in a developing nation like India.

## Conclusion

The result of this clinical study shows that Intracavitary brachytherapy with a dose of 9 Gy per fraction is non inferior to other schedules in term of local control and does not not result in increased toxicity.

## Data Availability

All data, models, or code generated or used during the study are available from the corresponding author by request.

## References

[1] Sung H, Ferlay J, Siegel RL, Laversanne M, Soerjomataram I, Jemal A, Bray F (2021). Global cancer statistics 2020 GLOBOCAN estimates of incidence and mortality worldwide for 36 cancers in 185 countries. Cancer J Clin.

[2] Mathur P, Sathishkumar K, Chaturvedi M, Das P, Sudarshan KL, Santhappan S, Nallasamy V, John A, Narasimhan S, Roselind FS, ICMR-NCDIR-NCRP Investigator Group (2020). Cancer statistics, 2020: report from national cancer registry programme India. JCO Global Oncol.

[3] Ncdirindia.org 2022 https://ncdirindia.org/NCRP/ALL_NCRP_REPORTS/PBCR_REPORT_2012_2014/ALL_CONTENT/PDF_Printed_Version/Chapter7_Printed.pdf.

[4] Narayanan V, Bista B, Sharma S (2012). External beam therapy in a four-field box technique with paclitaxel versus a two-field technique with cisplatin in locally advanced carcinoma cervix: a phase II monocentric trial. ISRN Oncology.

[5] Visanathan AN. Uterine cervix. In: Halperin EC, Wazer DE, Perez CA, Brady LW, editors.Perez and Brady’s Principles and Practice of Radiation Oncology. 6th ed. Philadelphia: Wolters Kluwer, Lippincott Williams Wilkins; 2013. 1: 1355–425.

[6] DiSilvestro PA, Walker JL, Morrison A, Rose PG, Homesley H, Warshal D (2006). Radiation therapy with concomitant paclitaxel and cisplatin chemotherapy in cervical carcinoma limited to the pelvis: a phase I/II study of the gynecologic oncology group. Gynecol Oncol.

[7] Hashemi FA, Akbari EH, Kalaghchi B, Esmati E (2013). Concurrent chemoradiation with weekly gemcitabine and cisplatin for locally advanced cervical cancer. Asian Pac J Cancer Prev.

[8] Patel FD, Kumar P, Karunanidhi G, Sharma SC, Kapoor R (2011). Optimization of high–doserate intracavitary brachytherapy schedule in the treatment of carcinoma of the cervix. Brachytherapy.

[9] Nag S, Erickson B, Thomadsen B, Orton C, Demanes JD, Petereit D, Society AB (2000). The American brachytherapy society recommendations for high-dose-rate brachytherapy for carcinoma of the cervix. Int J Radiat Oncol Biol Phys.

[10] Sood BM, Gorla G, Gupta S, Garg M, Deore S, Runowicz CD, Fields AL, Goldberg GL, Anderson PS, Vikram B (2002). Two fractions of high-dose-rate brachytherapy in the management of cervix cancer: clinical experience with and without chemotherapy. Int J Radiat Oncol Biol Phys.

[11] Ghosh S, Rao P (2016). High-dose-rate orthogonal intracavitary brachytherapy with 9 Gy/fraction in locally advanced cervical cancer: is it feasible??. J Obstet Gynecol India.

[12] Patel FD, Rai B, Mallick I, Sharma SC (2005). High-dose-rate brachytherapy in uterine cervical carcinoma. Int J Radiat Oncol Biol Phys.

[13] Novetsky AP, Einstein MH, Goldberg GL, Hailpern SM, Landau E, Fields AL, Mutyala S, Kalnicki S, Garg M (2007). Efficacy and toxicity of concomitant cisplatin with external beam pelvic radiotherapy and two high-dose-rate brachytherapy insertions for the treatment of locally advanced cervical cancer. Gynecol Oncol.

[14] Passi K, Kehwar TS, Mittal M, Singh B, Vashistha R, Gupta SJ, Yakhmi JV (2010). Effectiveness of two different HDR brachytherapy regimens with the same BED value in cervical cancer. J Contemp Brachytherapy.

[15] Hendry J, Jones GW, Mahantshetty UM, Sarria G, da Motta NW, Fidarova E, AbdelWahab M, Prasad RR, Polo A, Zubizarreta E (2017). Radiobiological analysis of outcomes using external beam radiotherapy plus high dose-rate brachytherapy (4x7 Gy or 2x9 Gy) for cervical cancer in a multi-institution trial. Int J Radiat Oncol Biol Phys.

[16] Ghosh S, Rao PB, Kotne S (2015). High dose rate brachytherapy in two 9 Gy fractions in the treatment of locally advanced cervical cancer-a south indian institutional experience. Asian Pac J Cancer Prev.

[17] Haie-Meder C, Pötter R, Van Limbergen E, Briot E, De Brabandere M, Dimopoulos J, Dumas I, Hellebust TP, Kirisits C, Lang S, Muschitz S (2005). Recommendations from gynaecological (GYN) GEC-ESTRO working group☆(I): concepts and terms in 3D image based 3D treatment planning in cervix cancer brachytherapy with emphasis on MRI assessment of GTV and CTV. Radiother Oncol.

[18] Schwartz LH, Litière S, De Vries E, Ford R, Gwyther S, Mandrekar S, Shankar L, Bogaerts J, Chen A, Dancey J, Hayes W (2016). RECIST 11—update and clarification: from the RECIST committee. Eur J Cancer.

[19] Protocol development CTEP. Ctep.cancer.gov https://ctep.cancer.gov/protocoldevelopment/electronic_applications/ctc.htm 2022.

[20] Rao BS, Das P, Subramanian BV, Jena A, Rashmi P, Konakalla VA, Jayasree K (2017). A comparative analysis of two different dose fractionation regimens of high dose rate intracavitary brachytherapy in treatment of carcinoma of uterine cervix: a prospective randomized study. J Clin Diagnostic Res.

[21] Perez CA, Grigsby PW, Castro-Vita H, Lockett MA (1995). Carcinoma of the uterine cervix I Impact of prolongation of overall treatment time and timing of brachytherapy on outcome of radiation therapy. Int J Radiat Oncol Biol Phys.

[22] Delaloye JF, Coucke PA, Pampallona S, De Grandi P (1996). Effect of total treatment time on event-free survival in carcinoma of the cervix. Gynecol Oncol.

